# The New Ethics of the New York State Medical Society

**Published:** 1882-04

**Authors:** 


### THE NEW ETHICS OF THE NEW YORK STATE MEDICAL SOCIETY.

The New York State Medical Society, at its recent meeting, adopted the following amendment to its code of ethics:

"Members of the Medical Society of the State of New York, and of the medical societies in affiliation therewith, may meet in consultation legally qualified practitioners of medicine. Emergencies may occur in which all restrictions should, in the judgment of the practitioner, yield to the demands of humanity."

We are not advised as to how this measure was carried through the association. We do not know how far it represents the whole state. Not a great many votes were recorded either as opposing or favoring the amendment, though it seems to have met earnest opposition.

It has been hinted that the measure was favored by specialists and others who desire to extend the field from which they may draw consultations. It may be that the case in England in which the physicians of Lord Beaconsfield consulted with a homœopathist turned the heads of some of the Americans who ape English customs. It may be that certain parties felt that, by the code of ethics, they were excluded from some cases in which a very rich consultation fee might otherwise be obtained. But at any rate the New York State Medical Society has placed itself in a very unenviable position. We imagine that some of the doctors there must feel the disgrace very keenly.

Consultation, in the true sense of the word, with a physician of a one-idea system is impossible. Most of the discussions have especially dealt with the homœopathists; but, by this code, no sect or school can be excluded. If a physician and homœopathist should endeavor to agree as to treatment, the consequences must be ridiculous.

The *Medical News* (Phila.) well says:

"The folly of a consultation between a regular physician, a homœopathist, and a botanical doctor, may be illustrated by an example. Suppose the case were one of violent cholera morbus, the regular physician would propose to cure promptly by the hypodermatic injection of morphia, the homœopathist would advise the third to the thirtieth dilution of veratrum, and the botanical doctor would urge No. 6. It would be simply impossible to reconcile the conflicting opinions, and the consultation would consist in the assertion of their respective principles." And we may add, all talk of consultation as to diagnosis and prognosis, but not as to treatment, is puerile.

But it is said that the educated homœopathists have abandoned the absurdity of the attenuated doses. Then let them confess it to the public as well as to themselves. Let them, then, cease to practice fraud by "trading upon a name." Let them declare themselves *doctors* and not "homœopathists." The whole profession knows the thing to be a fraud—not excepting the profession of New York. They have so proclaimed for years, and there has been nothing to produce a change of views. Why now endorse it? What then can be gained by such a course as the Society adopted? To quote again from the *Medical News:*

"To yield the point, of the right to consult with all kinds of irregulars who happen to be 'legally qualified practitioners,' is to admit that the regular medical profession has hitherto occupied a false position.

"To yield the point, is to admit that they who have maintained the truth of an exclusive dogma, and have practiced on a trade designation, were right, and those who opposed them were wrong."

"To yield the point, is to strengthen all irregular practitioners in their position of bigoted intolerance, and to confuse and dismay the disciples and friends of legitimate medicine."

It will not put a stop to the deceptions with which the irregulars are in the habit of imposing on the public. We cannot see how, as some have claimed, it can secure any better enactments before state legislatures. We do not see that there has been any necessity for *conciliating* the irregulars. If there was such necessity, then this step has proven a miserable failure. We quote from a homœopathic journal:

"It will thus be seen that the "Old School" has thrown down its "arms" and declared an end to hostilities within the Commonwealth so as it is concerned! The question now arises, how shall we of the "New School" conduct ourselves under the changed circumstances; shall we continue to fight or shall we except the situation in the temper in which it is offered?"

That is, we would translate, Must we forgive these erring brethren, and receive them into our fold? From another journal of about the same calibre:

"How wonderfully changed has the "medical world" become since Dr. Quain threw the bomb into the camp of professional intolerance from the sick-bed of Lord Beaconsfield! Bread and butter has been at stake for some time, and "old school physic" was ready for surrender. All that was needed was for some "general" to break the ranks and give himself over. See how the rest of the "sheep" follow! This is all right. Let the "shell" fall thick and fast, and rout the "ethical" fossils."

We find also that some of the secular press, entirely ignorant as they are of even the intention and objects of the code of ethics, and always ready to make cheap flings at what they term the hide-bound profession, are joining in the cackling and crowing such as we have quoted.

Now, if any one is enough of a knave to practice a fraud, or ignorant enough to engage in a one-idea so-called system, we do not see how we can prevent him. But we need not lend endorsement to what we know is either fraud or ignorance. We are glad the society in question does not *compel* its members to engage in such a farce as consultation with irregulars. We are glad a conscientious member may still maintain, individually, his self-respect. We believe, indeed, the end has not yet come even in New York. Surely the profession there will rebuke such action as was taken by these few men. We do not believe, as has been said, that New York, though she esteem herself the Empire State, can set her face against right and custom, and expect to find imitators in other States. And surely the society will not offer such an insult to the American Medical Association as would be implied in an attempt to send delegates thereto.

